# Flying under the radar: CDH2 (N-cadherin), an important hub molecule in neurodevelopmental and neurodegenerative diseases

**DOI:** 10.3389/fnins.2022.972059

**Published:** 2022-09-23

**Authors:** Zsófia I. László, Zsolt Lele

**Affiliations:** ^1^Momentum Laboratory of Molecular Neurobiology, Institute of Experimental Medicine, Budapest, Hungary; ^2^Division of Cellular and Systems Medicine, School of Medicine, University of Dundee, Dundee, United Kingdom

**Keywords:** N-cadherin, CDH2, adhesion, brain development, neurodegenerative diseases, neurodevelopmental diseases

## Abstract

CDH2 belongs to the classic cadherin family of Ca^2+^-dependent cell adhesion molecules with a meticulously described dual role in cell adhesion and β-catenin signaling. During CNS development, CDH2 is involved in a wide range of processes including maintenance of neuroepithelial integrity, neural tube closure (neurulation), confinement of radial glia progenitor cells (RGPCs) to the ventricular zone and maintaining their proliferation-differentiation balance, postmitotic neural precursor migration, axon guidance, synaptic development and maintenance. In the past few years, direct and indirect evidence linked CDH2 to various neurological diseases, and in this review, we summarize recent developments regarding CDH2 function and its involvement in pathological alterations of the CNS.

## Introduction

CDH2 is one of the most intensively studied of all the cadherins with thousands of references in PubMed. As a matter of fact, due to this heavy interest, it has become a bit of a bore after all these years, so when someone stands up at a conference introducing CDH2 as his or her subject of study, people tend to yawn, wish stronger for a coffee (or for a stronger coffee) and start reading the abstract of the next talk in the session. Yet somehow, year after year, this protein has been continuously able to surprise us by fulfilling yet another important developmental, physiological, or disease-related function. While aspects of CDH2 function during brain (particularly cortical) development have been studied extensively (Brayshaw and Price, 2016; Martinez-Garay, 2020; de Agustín-Durán et al., 2021), it has been largely overlooked as a potential factor in various neurodevelopmental and neurodegenerative diseases. In this review, we will summarize previous data regarding CDH2 function in neural development and disease with a particular emphasis on recent additions to the pathophysiological aspect which brought back this old, familiar protein into the limelight.

## The role of CDH2 in neural development

### Neuroepithelial integrity

*Cdh2* expression already appears during neural induction accompanied by a parallel decrease in *Cdh1* (E-cadherin) mRNA levels (Hatta and Takeichi, 1986). General disruption of *Cdh2* in the mouse results in early embryonic (E10) lethality due to cardiac developmental defects (Radice et al., 1997). At this point, the neural tube also displays an abnormally undulated phenotype, but further conclusions regarding its functions in CNS development could not be drawn. In contrast, nonsense mutations in the zebrafish *Cdh2* gene (Jiang et al., 1996; Lele et al., 2002), allow the mutant so-called *parachute* (*pac)*, named after the shape of the midbrain cross-section) to develop to a relatively mature stage before becoming lethal (i.e., 48 h post-fertilization, at which point the wild-type zebrafish larvae are hatched and display complex behaviors like swimming and feeding). This is probably due to the combination of quick CNS development and the small size of the embryo which allows a sufficient supply of nutrients from the yolk sac and oxygen *via* simple diffusion into the embryonic tissues. This makes the cardiac function largely irrelevant in this stage of development during which neural induction, patterning, neurulation, and most of the early differentiation processes are already completed. In *pac* mutants, the epithelial structure of the dorsal neural tube gets disrupted in the dorsal part of the midbrain-hindbrain region (Jiang et al., 1996). Similar symptoms also appear in chick embryos after the application of CDH2-function blocking antibodies (Gänzler-Odenthal and Redies, 1998) indicating that CDH2-based adherens junctions also maintain neuroepithelial integrity in Amniotes.

### Neurulation and neural crest development

CDH2 is essential for both zebrafish and mouse neurulation despite the fact that neural tube formation follows a different route in these organisms (convergent-extension movements of neural plate cells followed by secondary cavitation vs. direct neural tube formation, respectively). In the zebrafish loss-of-function *Cdh2* mutant *pac*^*fr*7^, convergent-extension movements of neural plate cells are disrupted thereby delaying the migration of neural plate cells toward the midline (Lele et al., 2002). As a result, these cells get excluded from the forming neural rod. In the mouse, cardiac rescue of the *Cdh2−/−* animals allows development to proceed further than in the full knockout and in these animals, closure of the anterior neuropore never occurs also indicating a role (albeit a spatially restricted one) for CDH2 in neurulation (Radice et al., 1997; Luo et al., 2001).

**FIGURE 1:**
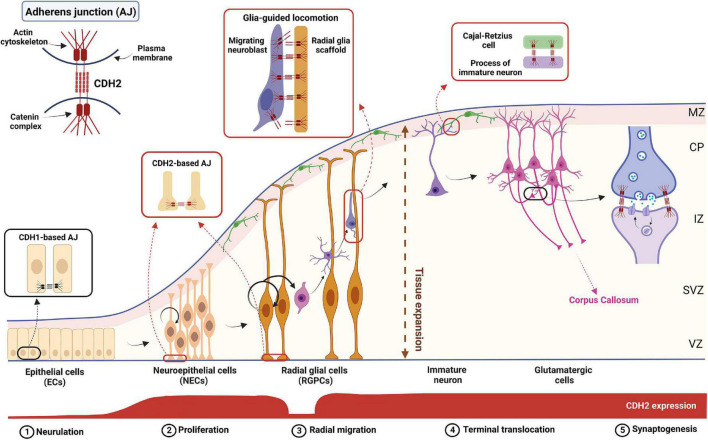
The complex role of CDH2 during cortical development. Schematic diagram showing the different stages of cortical development. CDH2-based adherens junctions appear after neurulation and are present in both NECs and RGPCs. After asymmetric cell division in the ventricular zone (VZ), newborn, non-radial glia-fated daughter cells delaminate from the RGPCs and undergo radial glia-guided locomotion through the subventricular and intermediate zones (SVZ and IZ, respectively) utilizing CDH2 connections. Once the immature neuron reaches the cortical plate (CP), CDH2 connections between the leading process and a Cajal-Retzius cell in the marginal zone (MZ) are formed which helps to reach and maintain the neuron’s final position in the cortical plate. Further on, during synaptogenesis homophilic CDH2 binding help neuronal outgrowth and synaptogenesis to build up the proper cortical circuits. Figure was created using Biorender.com.

Emergence of neural crest (NC) cells from the dorsal neural tube occur simultaneously with neural tube closure. As mentioned above, the closure also requires the presence of CDH2, the main component of adherens junctions keeping neuroepithelial cells to each other (Figure 1). Thus, it is no surprise that escaping the closing neuroepithelium requires a transient decrease of CDH2 expression in NC cells in favor of cadherins with weaker binding ability, CDH6, then CDH7 (Nakagawa and Takeichi, 1995, 1998; Coles et al., 2007; Taneyhill et al., 2007; Park and Gumbiner, 2010; Schiffmacher et al., 2014). Repression of CDH2- mediated adhesion is prompted by BMP4 signal-induced ADAM10 activity (Sela-Donenfeld and Kalcheim, 1999). The C-terminal fragment of CDH2 enters the nucleus and promotes CyclinD transcription and neural crest delamination (Shoval et al., 2006).

Despite the transient decrease of CDH2 levels, the protein has an important function later in the collective migration of neural crest cells in Anamniotes. CDH2-based connections not only provide adhesive force between migrating cells, but are also actively involved in migration *via* treadmilling (Peglion et al., 2014). In addition, intercellular CDH2-dimerization establishes the cells’ polarity by altering the actin cytoskeleton *via* RhoA and Rac1 which in turn enables their directed migration toward attracting signals such as SDF1a/CXCL12 (Theveneau et al., 2010; Taneyhill and Schiffmacher, 2017; Szabó and Mayor, 2018).

### Radial glia functions and neuronal differentiation during cortex development

Neuroepithelial cells convert to radial glia progenitor cells (RGPCs) in the mouse pallium around E10, a process hallmarked by the loss of tight junctions and extension and thinning of the basal process (Aaku-Saraste et al., 1996). These cells have a dual function during cortical development: by asymmetric cell division they generate an RGPC-fated and a non-RGPC-fated cell, the latter could become an intermediate progenitor cell (IPC) or a fate-committed neuroblast. In addition, the basal process of RGPCs extends to the basal (pial) surface providing a scaffold for the fate-committed neuroblasts to migrate on (Noctor et al., 2004). In rodents, RGPCs in the dorsal part of the telencephalon (the pallium) generate mostly excitatory neurons, while birth of the inhibitory neurons occurs primarily in the ganglionic eminences and the preoptic area of the subpallium (Anderson et al., 1997). Loss of *Cdh2* in RGPCs interferes with the most important processes during cortical development including proliferation, differentiation and cell migration (Kadowaki et al., 2007; Gil-Sanz et al., 2014; László et al., 2020a). These together manifest in severe lamination defects of the mouse cortex (Kadowaki et al., 2007; Miyamoto et al., 2015) similar to what was found in the zebrafish retina earlier (Erdmann et al., 2003; Malicki et al., 2003; Masai, 2003). CDH2-based adherens junctions are also essential for maintaining the stemness of RGPCs *via* the promotion of β-catenin- and Notch signaling (Zhang J. et al., 2010; Hatakeyama et al., 2014). Loss of *Cdh2* disrupts not only the radial glia scaffold which hinders the radial migration of neuroblasts, but also the apical cell-to-cell contacts between RGPCs resulting in their dispersion (Kadowaki et al., 2007; Gil-Sanz et al., 2014; László et al., 2020b). In addition, it also promotes premature differentiation (hence a decrease in the number) of PAX6-positive RGPCs into TBR2-positive intermediate progenitor cells (IPCs) and TBR1-positive neurons (Zhang J. et al., 2010). In contrast, *Emx1-Cre*-induced loss of *Cdh2* increases the proliferation of precursors resulting in severe cortical heterotopia (Gil-Sanz et al., 2014). The explanation for this contradiction might lie within the difference in applied methods (shRNA knockdown vs. tissue-specific KO) or in the timing of the *Cdh2*-loss. The electroporation experiment was carried out at E12.5 while the knockout study used the *Emx1-Cre* which acts quite early in pallial cortical differentiation (E9.5-E10.5) therefore the pro-proliferative effect might be due to the loss of *Cdh2* in neuroepithelial cells. Alternatively, it is feasible that there is a developmental time-specific usage of the intracellular α-catenins. This is supported by the fact that only αE-catenin can be found in the ventricular zone of the early E12 cortex, αN-catenin appears only later and even then, only in the subventricular zone (Uchida et al., 1996; Ajioka and Nakajima, 2005; Stocker and Chenn, 2009). In addition, a hyperplasia, similar to what was found in the *Emx1-Cre*-mediated *Cdh2 −/−* embryos, has been only reported in *αE-catenin*, but not in *αN-catenin* knockout animals (Park et al., 2002; Lien, 2006; Schmid et al., 2014).

CDH2 is also essential for the proper formation of inhibitory neurons born in the subpallium. Although much less publicized than its role in the development of pallial neurons, it has been demonstrated that CDH2 plays a similar role in the subpallial ventricular zone. Loss of *Cdh2* resulted in severe disorganization of the medial ganglionic eminence (MGE) proliferating zone (Luccardini et al., 2013). The number of phosphohistone 3-positive mitotic progenitor cells decreased which in turn resulted in less interneuron precursors reaching the cortex. These data resemble the results found in the pallium although the number of intermediate progenitors in the MGE has not been examined (Zhang J. et al., 2010).

Importantly, pathophysiological disruption of classic cadherin-based adherens junctions (i.e., CDH1 and CDH2) between RGPCs and their evoked random dispersal to upper layers provokes protective suicidal apoptosis in the embryonic cortex called developmental anoikis (László et al., 2020b). This prevents random migration of proliferating progenitors in the cortex which in turn leads to heterotopia and provides an explanation for the relatively low prevalence of these diseases considering the astronomical number of cell division events. It is important to note that the exact cadherin involved in this process has not been identified yet, hence the possibility of compensatory action from other classic cadherins (i.e., CDH1 or even some type II cadherins) which are expressed in the VZ of the developing neocortex, cannot be excluded (Rasin et al., 2007; Lefkovics et al., 2012). Nevertheless, a previous study examining the loss of CDH1 caused by overexpression of its repressor *Scratched* did not find elevated cell death levels in the embryonic cortex, indicating that the cell death requires the loss-of CDH2, or both CDH1 and CDH2 (Itoh et al., 2013).

### Role of CDH2 in postmitotic neural precursors

The role played by CDH2 in neuronal precursor migration has been studied in detail both in pallial and subpallial neurogenesis. In the pallium, after asymmetric cell division, the non-radial glia-fated daughter cell needs to downregulate CDH2 in order to lose its cell-cell connections which allows the escape from the ventricular zone adherens junction belt (Figure 1). This downregulation is evoked by the NGN2-activated FOXP2/4 transcriptional repressor (Rousso et al., 2012). Loss of *Cdh2* is also essential for the apical abscission process and consequent dismantling of the primary cilium in the migrating cell (Das and Storey, 2014).

Later during neuroblast migration, however, the presence of CDH2 becomes essential again for the multipolar-bipolar transition occurring at the SVZ prior to glial-guided migration of neuroblasts toward the cortical plate (Franco et al., 2011; Jossin and Cooper, 2011; Gil-Sanz et al., 2013). The following glial-guided locomotion also utilizes CDH2 anchoring at the front and recycling at the back of the migrating cell to promote directed migration (Shikanai et al., 2011). The recycling mechanism involved in this is discussed in detail below as it has significant medical relevance. Finally, cadherin function is also necessary for the last part of radial migration called terminal translocation when the migrating neuron extends a process toward the pial surface and after connecting there, utilizes somal translocation to reach its final destination within the cortical plate (Nadarajah et al., 2001). This is demonstrated by the postmitotic cell-specific *Dcx* promoter-driven expression of dominant negative CDH2 which resulted in migration arrest just prior to the cortical plate entry (Franco et al., 2011).

In the subpallium, CDH2 also plays a substantial role in the migration of postmitotic interneuron (IN) precursors to the cortical plate. CDH2-mediated migration of precursors is 7-times faster on CDH2-coated when compared to laminin-coated surface. This is due to strongly coordinated nuclear and centrosome movements which are disrupted in *Cdh2−/−* precursors (Luccardini et al., 2015). Loss of *Cdh2* in postmitotic cells of the MGE results in delayed migration due to randomized localization of the centrosome within the cell. In turn, this leads to the loss of both the leading process and the polarized phenotype of migratory interneuron precursors in general (Luccardini et al., 2013). Unlike in the proliferating progenitor cells, postmitotic knockout of *Cdh2* does not affect proliferation and cell death rates in the ganglionic eminences (László et al., 2020a). Tangential migration of the affected cells, however is delayed and due to this, some of the precursors reach the pallium too late and cannot enter the cortical plate. Consequently, they are eliminated by the developmental apoptosis process which occurs at the end of the first postnatal week (Southwell et al., 2012; Wong et al., 2018). Remarkably, however, this effect was cell-type specific. The adult somatosensory cortex of *Dlx5/6Cre:Ncad*^fl/fl^** mice showed a strong reduction in CALB2 (calretinin)- and/or SST (somatostatin)-positive interneurons while the number of parvalbumin-positive INs did not change (László et al., 2020a). The molecular mechanism behind this cell-type-specific requirement of CDH2 is currently unclear, although it must be mentioned here that expression of both *Cdh2* and *Calb2* are highly regulated by the Aristaless (*ARX*) transcriptional repressor, an important factor in interneuron differentiation (Kitamura et al., 2002; Friocourt et al., 2008; Quillé et al., 2011).

### The reelin signaling pathway and the mechanism of CDH2 action in pallial cortical migration

*Reelin* is a secreted glycoprotein produced by Cajal-Retzius cells which forms a decreasing gradient in the pial-ventricular direction within the embryonic cortex (Soriano and Del Río, 2005; Sekine et al., 2011). Spontaneous recessive mutation of its gene produces the mouse mutant *Reeler* which is characterized by inverted cortical lamination (D’Arcangelo et al., 1995; Ogawa et al., 1995). Neuroblasts undergoing their final stage of migration to the cortical plane *via* terminal translocation are expressing the receptors of Reelin, the very low-density lipoprotein receptor (VLDLR) and the apolipoprotein receptor E2 (ApoER2; Lane-Donovan and Herz, 2017; Dlugosz and Nimpf, 2018). Loss of these receptors produces a similar inverse cortical phenotype found in the *Reeler* mutant (Trommsdorff et al., 1999; Hack et al., 2007). Receptor activation initiates the phosphorylation of Disabled-1 (DAB1) which in turn causes the stabilization of the cytoskeletal protein, cofilin, and the recruitment of integrins to the leading process of the migrating cell. This promotes the establishment of homophilic CDH2 connections between Cajal-Retzius cell and the migrating glutamatergic cell which is essential for terminal translocation the induction of neurite arborization and synaptogenesis (Franco et al., 2011; Gil-Sanz et al., 2013; Matsunaga et al., 2017; Jossin, 2020). For further details of Reelin signaling and function during brain development, the reader is referred to a recently published excellent review (Jossin, 2020).

### Regulation of membrane-bound CDH2 protein levels

As we have seen the level of membrane-bound CDH2 is of fundamental importance for cell-cell adhesion during cortical development. Levels of CDH2 must be decreased during neural crest cell exit from the closing neural tube. During neurogenesis, the non-radial glia-fated daughter cell also must decrease their CDH2 levels in order to escape from the ventricular zone. Consequentially, active regulation of CDH2 protein levels represents a possibility to control cell migration. This decrease, however, is transient, as both radial glia-directed migration and the terminal translocation step require the presence of the protein. This tight control of CDH2 protein levels at the cell surface is carried out by two distinct mechanisms: endosomal recycling and proteolytic cleavage.

Endosomal recycling is carried out *via* different Rab-GTPases providing spatial and temporal regulation of CDH2 levels during different phases of radial migration. During glia-guided locomotion, the accumulation of the protein in the migrating precursor cell identifies the leading process which maintains a proper attachment to the radial glia scaffold. In contrast, CDH2 is endocytosed at the posterior end of the cell and these two parallel processes create a treadmilling effect moving the cell along the scaffold. Stable and dynamic locomotion of the cells is ensured by the coordinated action of endocytic vesicle-associated Rab-GTPases such as Rab5, Rab7, Rab11, and Rab23 (Kawauchi et al., 2010; Shikanai et al., 2011; Hor and Goh, 2018). CDH2 is internalized through clathrin- and dynamin-mediated Rab5-dependent endocytosis which could be followed by lysosomal degradation or Rab11-dependent recycling to the plasma membrane (Kawauchi et al., 2010; Shieh et al., 2011). At the final phase of radial migration, called terminal translocation, CDH2 is internalized by a Rab7-dependent pathway which allows the leading process to detach from the radial fiber and form connections to the Cajal-Retzius cells in the marginal zone (MZ, Chai et al., 2009; Kawauchi et al., 2010). This phenomenon is induced by the large extracellular glycoprotein, Reelin as discussed above (Jossin and Cooper, 2011; Matsunaga et al., 2017).

Proteolytic cleavage of CDH2 is also an important way of regulating its cell surface levels which is carried out sequentially by ADAM10 (A Disintegrin and metalloproteinase domain-containing protein 10) and Presenilin 1 (PSEN1) acting as α- and γ-secretases, respectively (Baki et al., 2001; Marambaud et al., 2003). ADAM10 is a member of the α-disintegrin-and-metalloprotease family sometimes also referred to as MADM or Kuzbanian Protein Homolog after the name of the Drosophila mutant. Loss of ADAM10 results in embryonic lethality with some features resembling *Cdh2-/-* embryos, including E9.5 embryonic lethality with defective somite, heart and vascular development and CNS abnormalities (Hartmann et al., 2002). Radial glia-specific disruption of *Adam10* results in perinatal lethality due to vascular hemorrhages in the brain. Importantly, The CTF1 generation of CDH2 was severely reduced in the KO animals. The affected mice also feature disrupted cortical lamination and decreased subpallium size, particularly the caudal ganglionic eminence (Jorissen et al., 2010). In addition, proliferation levels were also decreased during later stages (from E15 onward) of cortical development. Despite the shared features and fate of *Adam10−/−* embryos to *Cdh2−/−* animals, only two other ADAM10 targets, NOTCH and APP (Amyloid precursor protein) were analyzed in this study.

As discussed above, metalloproteases like ADAM10 directly regulate the level of CDH2 at the cell surface (Marambaud et al., 2003; Reiss et al., 2005). Interestingly, extracellular cleavage of CDH2 is also prevented by loss-of *Rab14*. This is due to the trapping of ADAM10 in an endocytic compartment thereby decreasing its levels in the cell membrane. As a result, CDH2-shedding is impaired, and its levels are increased on the cell surface inhibiting cell migration. Accordingly, this effect of *Rab14*-loss could be reverted by siRNA knockdown of CDH2 levels (Linford et al., 2012). It is important to emphasize that the migration assay was carried out on A549 lung carcinoma (i.e., epithelial) cells. Whether a similar effect is also induced in neurons is yet to be seen. Furthermore, ADAM10-mediated shedding liberates secondary messenger molecules from the intracellular complex leading to gene-expression changes (i.e., β-catenin) and cytoskeletal reorganization (*via* p120catenin). Finally, it is essential to note that knock-in mice expressing a cleavage-resistant form of CDH2 did have any early neural development changes indicating that ADAM10-mediated cleavage is not important during these processes (Asada-Utsugi et al., 2021).

### CDH2 in neurite outgrowth, axon specification and guidance

Neurite outgrowth is one of the earliest and most thoroughly studied developmental processes to be promoted by CDH2 (Neugebauer et al., 1988; Tomaselli et al., 1988; Bixby and Zhang, 1990; Riehl et al., 1996). The role of CDH2 in axonal and dendritic development, however, already begins at the polarization of the postmitotic cell after asymmetric division (Pollarolo et al., 2011). CDH2 concentration in the postmitotic cell promotes centrosome recruitment and the site of first neurite formation *via* PI3K signaling (Gärtner et al., 2012). It has been demonstrated that in the mouse embryonic cortex, NUMB, and NUMBL, two factors inherited asymmetrically by the progenitor but not the postmitotic cell, are co-localized in the apical extension with cadherin-based adherens junctions and help to maintain the apicobasal polarity of the RGPC-fated daughter cell. Coincidentally, disruption of *Numb* and *Numbl* results in the loss of cadherin connections and consequentially polarity in the progenitor cell (Rasin et al., 2007). As we discussed earlier, CDH2-based connections to radial glia fibers during glia-guided locomotion are essential for proper neuronal migration. Even as the postmitotic neuron migrates, however, dendrite vs. axon polarity is already being established in the cell in a CDH2-dependent manner. Recently, Kaibuchi and colleagues demonstrated that axon specification occurs at the opposite side of RGPC-neuron cadherin connections. Disruption of cadherin-based AJs also causes the loss of polarity and axon specification *via* interference with Rho and Rac1 kinase functions (Xu et al., 2015).

CDH2-dependence of axon guidance and targeting has been studied extensively. Without getting into too much detail, CDH2 has been demonstrated to be involved in these processes from Drosophila to mammals (Matsunaga et al., 1988; Riehl et al., 1996; Clandinin and Zipursky, 2002; Lele et al., 2002; Treubert-Zimmermann et al., 2002; Hirano and Takeichi, 2012; Sakai et al., 2012; Jontes, 2018; Yamagata et al., 2018; Sanes and Zipursky, 2020). In this respect, the interaction of CDH2 with the SLIT-ROBO signaling is of particular importance. Interestingly this pathway has been implicated previously in a set of developmental processes (e.g., progenitor proliferation and differentiation, interneuron migration, dendrite development, midline crossing of axons) very similar to those also requiring CDH2 function (Plump et al., 2002; Whitford et al., 2002; Andrews et al., 2007; Plachez et al., 2008; Borrell et al., 2012). Yet detailed interaction between CDH2 and SLIT-ROBO signaling has only been described in detail for axon guidance. In summary, activation of ROBO *via* its repulsive guidance cue partner SLIT recruits the Abelson kinase (ABL) which in turn binds the β-catenin-CDH2 complex *via* the linker molecule CABLES. ABL then phosphorylates and frees β-catenin from the adhesion complex which enters the nucleus and promotes the transcription of its targets. Consequently, this process also results in the weakening of CDH2-mediated adhesion and actin cytoskeleton rearrangement, two important steps in growth cone steering (Rhee et al., 2002, 2007). It is very tempting to propose that closer examination of CDH2-SLIT-ROBO interaction in the other, above-mentioned neurodevelopmental processes could yield important results and help to understand their molecular nature.

### CDH2 in synaptogenesis, synapse maintenance and synaptic function

Considering the reversibility of CDH2-based cell-cell interactions, it is not surprising that this molecule also plays a critical role during synaptogenesis and synaptic plasticity. Synapse formation starts in mid-gestation in humans and perinatally in rodents. It is a strictly regulated process influenced by different autocrine and paracrine signals which lead to the formation of the morphologically distinct pre- and postsynaptic regions divided by the synaptic cleft (Li et al., 2010; Budday et al., 2015). The synaptic regions of the two neurons are attached *via* bridge-like adhesion proteins between the two sides. The first, albeit indirect indication that CDH2 is expressed in the synapse was published in 1996 showing synaptic localization of αN-catenin which preferentially binds to CDH2 (Uchida et al., 1996). Later, several studies provided direct evidence using immunohistochemistry and confocal, as well as electron microscopy demonstrating that CDH2 protein appears in both the pre- and postsynaptic membrane. Its distribution is random at first within the synaptic region but as the synapse matures, the CDH2 dimers are gradually restricted to focal points within the synapses (Togashi et al., 2002; Elste and Benson, 2006; Yam et al., 2013).

Cytoskeletal structure and activity-dependent actin reorganization are key elements of both synapse formation and synaptic transmission. Adhesion complexes are usually tightly embedded in the plasma membrane and also hold a strong connection to the cytoskeleton *via* their intracellular part which binds several secondary catenin proteins in a multimolecular complex which creates a multifunctional protein hub (reviewed in Mège and Ishiyama, 2017). CDH2-based junctions, synaptic morphology and synaptic function form a mutually interdependent feedback loop where stronger synaptic activity strengthens synaptic adhesion and spine morphology and vice versa, strong synaptic adhesion leads to maturation of dendritic spines and elevated synaptic transmission (Tanaka et al., 2000; Togashi et al., 2002; Okamura et al., 2004; Bozdagi et al., 2010; Mendez et al., 2010). Importantly, the promotion of synaptic adhesion stabilizes the actin cytoskeleton of the dendritic spine, while loss of adhesion triggers destabilizing cytoskeletal changes. Using live-cell imaging and computer simulations, researchers demonstrated that ablation of CDH2 leads to spine shrinkage which highly depends on the reorganization of its actin cytoskeleton (Mysore et al., 2007; Chazeau et al., 2015). Accordingly, Mendez et al. (2010) showed that long-term potentiation promoted CDH2 cluster formation in stimulated spines. In contrast, utilizing an extracellular domain-lacking mutant CDH2 (Δ390-Ncad) caused not only the spine shrinkage but also a decrease in the size of its postsynaptic density (PSD) and consequently, LTP formation.

These transsynaptic CDH2-CDH2 interactions provide not only mechanical support but also evoke both synaptogenesis and synaptic maturation which can help to hig-hlight the functional site of synaptic transmission. To initiate this process, the localization of CDH2 in the axonal growth cone or the developing spine is crucial (Basu et al., 2015). Any disruption of CDH2 or its associated molecules in the adhesion complex leads to spine ablation and reduction in key synaptic proteins such as synaptophysin or PSD95 (Togashi et al., 2002; Elia et al., 2006). Similarly, asymmetric expression of CDH2 in the pre- and postsynaptic cells results in synapse elimination and axon retraction (Pielarski et al., 2013). It has been shown that phospholipase D1 (PLD1) promotes dendritic spine development and increased synaptic adhesion and strength by elevating CDH2 levels in the cell membrane. This occurs *via* inhibition of the ADAM10 metalloprotease, which normally cleaves the extracellular domain of CDH2 (Luo et al., 2017). In addition, a year later the same laboratory found that the protein kinase D1 (PDK1) can phosphorylate the intracellular loop of CDH2 thereby promoting its membrane localization (Cen et al., 2018). Accordingly, disruption of PDK1-CDH2 interaction leads to reduced synapse numbers and impaired LTP formation (Cen et al., 2018). Finally, they demonstrated that these processes are all connected and part of the same pathway. PLD1 activates PDK1 which then regulates CDH2 and promotes spine morphogenesis (Li et al., 2019). In another study, Yamagata and colleagues found that other adhesion proteins such as the presynaptic neurexin1β require intercellular CDH2 dimerization to initiate proper postsynaptic differentiation (Yamagata et al., 2018).

Surprisingly, *in vitro* neuronal differentiation of embryonic stem cells from homozygous CDH2 knock-out blastocyst or conditional ablation of CDH2 from excitatory synapses (Bozdagi et al., 2010) did not replicate the aberrant synapse morphology described before but showed altered AMPA-mediated miniature EPSCs and reduced high-frequency stimulation-evoked vesicular release (Jüngling et al., 2006). The authors also found slower vesicular refill and turnover which in turn led to alterations in short-term plasticity. Further investigations revealed that homophilic CDH2 binding enhances vesicular endocytosis in a release-activity-dependent manner thereby coordinating and balancing the two processes in cooperation with Neuroligin1 (Stan et al., 2010; Van Stegen et al., 2017). In addition, this strong adhesion also maintains receptor recycling and stabilization at the postsynaptic region which also highlights its transsynaptic functions. It has been shown, that the GluA2 subunit of postsynaptic AMPA receptors directly interacts with CDH2 extracellular domain in both *cis* (i.e., CDH2 within the same cell) and trans (i.e., CDH2 in the presynaptic cell) manner. This heterophilic binding helps the lateral diffusion of AMPA receptors, which determines the efficacy of excitatory postsynaptic potential and strengthens synaptic morphology (Saglietti et al., 2007). In another study, researchers demonstrated that the interaction of CDH2 and GluA2 leads to cytoskeletal changes by the activation of the actin-binding protein, cofilin. This data also revealed that electrophysiological changes determine the postsynaptic morphology and receptor availability in a CDH2-dependent manner (Zhou et al., 2011). Altogether, these precise molecular changes unravel the task of CDH2 in regulating the activity of the whole network which affects memory formation and behavior (Schrick et al., 2007; Asada-Utsugi et al., 2021).

### CDH2 and neuronal cell death

There is one more cellular process that can be linked to CDH2, and that is cell death. In physiological conditions, proper cell-to-cell adhesion perpetuates downstream pro-survival pathways and maintains cell growth and health. *In vitro* evidence demonstrates that CDH2-based adherens junctions stabilize the expression of several anti-apoptotic molecules, such as BCL-2 or support anti-apoptotic pathways mediated by Erk1/2 MAP kinase (Tran et al., 2002; Lelièvre et al., 2012). During certain pathophysiological conditions like fibrosis or metastasis. epithelial cells undergo an epithelial-mesenchymal transition (EMT) which is accompanied by decreased CDH1 and elevated CDH2 expression. CDH2-mediated intercellular connections not only promote the cytoskeletal transition to a migratory phenotype but also act as pro-survival factors (Ko et al., 2012; Geletu et al., 2022). Loss of cell to extracellular matrix adhesion during EMT triggers a specific type of programmed cell death pathway called anoikis to prevent anchorage-independent cell proliferation (Paoli et al., 2013). Some of the metastatic cells, however, can develop an anoikis-resistant phenotype which is a key step to successful cancer metastasis. Interestingly, an identical but physiological process also takes place during cortical development. The establishment of cortical layering highly depends on the asymmetric proliferation of radial glial progenitor cells. Following cell division, the non-radial glia-fated cell delaminates by downregulating its connection to the neighboring cells at the apical and to the ECM at the pial surface while also changing its morphology in preparation for radial migration (Taverna et al., 2014). The molecular mechanism of this process strongly resembles that of classic EMT. Yet despite becoming anchorage-independent, these cells can survive (Singh and Solecki, 2015) which means that there is no anoikis-like protective mechanism during this process, or if there is, the non-radial glia-fated daughter cell can somehow escape it. On the other hand, the daughter cell that becomes a proliferative RGPC remains anchored and preserves its apico-basal polarity. Needless to say, that free migration of RGPCs would present an increased risk for brain malformations such as various forms of heterotopias. The frequency of these diseases, however, is fairly low despite the millions of cell divisions during cortex development, indicating the presence of a protective anoikis-like cell death mechanism. Indeed, it has been recently demonstrated, that the ablation of cadherin-based adherens junctions by molecular methods and certain chemotoxic (ethanol) insults during cortical development both led to intrinsic, caspase3-mediated cell death (László et al., 2020b).

To further support the protective function of adherens junctions, elevated cell death has been described after the ablation of various other components of the AJ and the actin cytoskeleton. Due to spatial constraints, the reader is referred to a recently published excellent review on this topic (Veeraval et al., 2020).

## The role of CDH2 in neuropsychiatric diseases

**FIGURE 2:**
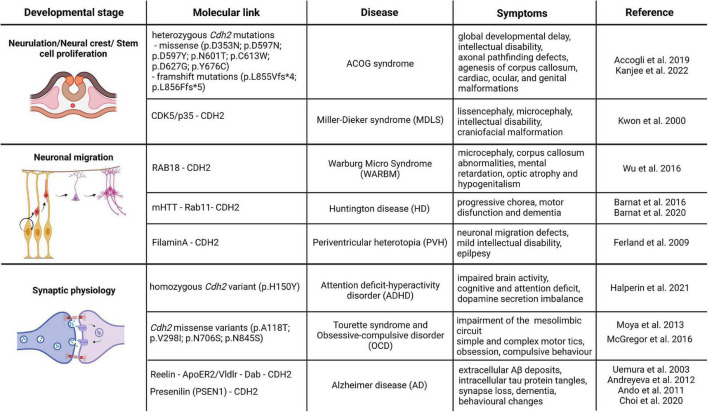
*CDH2-linked* neurodevelopmental and neurodegenerative diseases. The schematic drawings in the figure represent the different developmental processes associated with *CDH2* function and the associated diseases based on the genetic or direct molecular link. Image was created using Biorender.com.

Based on the overwhelming evidence from various loss-of-function models, CDH2 is unambiguously one of the most important cell adhesion molecules during brain development with important roles in neurulation, neuronal proliferation, differentiation and migration, axon guidance, synaptogenesis and synaptic maintenance. Despite this, *Cdh2* has not been directly linked genetically to any human neurodevelopmental disorders until recently. Below, we summarize various neurological diseases linked to *Cdh2* in the last 10 years and provide details of the underlying molecular mechanisms where it is available (Figure 2). We only discuss diseases due to direct mutations of the *Cdh2* gene or mutations which cause defective regulation of the protein. Other diseases which have only a second-degree connection to CDH2 such as those affecting the various molecules of the actin cytoskeleton (with one exception) or tubulinopathies will not be included due to space constraints.

### ACOG-syndrome

In 2019, Accogli et al. reported *de novo* heterozygous pathogenic *Cdh2* variants in 9 individuals. The six different missense mutations affected the extracellular domain of the protein, while the two distal frameshift mutations were found in the cytoplasmic region. Patients had various symptom combinations including global developmental delay, intellectual disability, axonal pathfinding defects, cardiac, ocular, and genital malformations. In order to summarize this wide spectrum of symptoms the collective term ACOG syndrome was created (agenesis of corpus callosum, axon pathfinding, cardiac, ocular, and genital defects; Accogli et al., 2019; Kanjee et al., 2022). These symptoms correlate well with some features of loss-of-CDH2 function models in mice (Riehl et al., 1996; Redies, 1997; Kadowaki et al., 2007; Oliver et al., 2013; Piprek et al., 2019) and other vertebrates (Gänzler-Odenthal and Redies, 1998; Lele et al., 2002; Masai et al., 2003; Rebman et al., 2016). Of the various mutations, those that are located on the extracellular part of CDH2 all resulted in weaker adhesion than created by the wild-type protein. In particular, Asp353Asn mutation affects one of the Ca^2+^-binding sites important for trans dimerization and consequently cell-to-cell adhesion (Vendome et al., 2014). The rest of the extracellular domain mutations probably also provide weaker adhesion due to the not perfect sterical alignment of the extracellular cadherin domains during cis and trans dimerization. The frameshift mutations affecting the intracellular part of the molecule obviously prevent proper binding of p120 and/or β-catenin to CDH2 thereby interfering with its interaction with the actin cytoskeleton.

Attention deficit and hyperactivity disorder (ADHD), Obsessive compulsory disorder (OCD), Tourette syndrome (TS), Autism spectrum disorder (ASD).

These diseases have often been shown to express comorbidity, so they will be discussed together.

### Attention deficit and hyperactivity disorder

Very recently, Halperin and colleagues reported the first familial *Cdh2* mutation connected to attention-deficit hyperactivity disorder (ADHD). Three siblings of the subject were diagnosed with ADHD in early childhood, two of them are non-identical twins who were born prematurely. Performing whole-genome sequencing, researchers found a single homozygous variant in the locus of c.355 C > T; p.H150Y in *Cdh2* gene, affecting the first full transcript variant. This mutation results in a single amino acid change in a non-organized loop structure between the first extracellular domain of the protein and the pro-domain. This region is indispensable for further protease modification by proteolytic enzymes such as the FURIN protease (Halperin et al., 2021). The mutant tyrosine changes the conformation of the binding site, therefore, inhibiting the maturation of the protein. Generating a unique animal model which has this new familial mutation, they found that transgenic animals replicate the human ADHD behavioral phenotype. Moreover, further immunohistochemical analysis revealed that familial *Cdh2* mutation decreases the size of the presynaptic vesicle cluster and changes the excitability of the neurons in the prefrontal cortex and hippocampus. Importantly, this change was cell-type-specific, affecting the dopaminergic neurons in the VTA which project to higher cognitive areas, highlighting the importance of CDH2 function during the previously ignored midbrain development and connectivity. The reduced tyrosine hydroxylase expression and consequent dopamine level drop in the ventral midbrain and prefrontal cortex indicates a compromised reward mechanism which could explain the increased attention-seeking behavior of the affected children (Halperin et al., 2021).

### Obsessive compulsory disorder

In 2009 Dodman et al. (2010) carried out a genome-wide association study with fine mapping of the Chromosome 7 region involved in dogs phenotyped for signs of canine compulsory disorder CCD. The most significantly associated single nucleotide polymorphism (SNP) was located within the *Cdh2* gene. In addition, targeted exon sequencing analyses found SNPs within *Cdh2* linked to both increased and reduced risk to develop OCD (McGregor et al., 2015) strongly indicating the involvement of CDH2 in the disease.

### Tourette-syndrome

There are two whole exome sequencing studies involving TS patients which identified variants within the *Cdh2* gene (Moya et al., 2013; Nazaryan et al., 2015). The first study could not establish enough evidence for an association between the mutations and TS but concluded that CDH2 and cadherins in general are interesting genes to study in the future as plausible contributors to TS and OCD (Moya et al., 2013). The second study could not find the SNP in their cohort described in the previous study but combining their data with other studies concluded that the variant described previously, albeit extremely rare, is indeed associated with both TS and OCD (Nazaryan et al., 2015). Furthermore, they also described a single nucleotide variant which occurred in a patient with combined symptoms of TS, OCD, ASD and ADHD. It is important to note, however, that both studies have used a relatively small cohort (N = 219 and N = 320), but these results justify further larger-scale GWA studies in this direction.

### Autism spectrum disorder

In a recent study, Liu and colleagues reanalyzed two previous large-scale literature studies involving ASD and OCD research data to identify novel target genes for future GWA studies (Liu et al., 2019). For the genes being predicted for both diseases, a protein-protein interaction literature search was also carried out. The co-occurrence rate for genes linked to both diseases was found to be 6.4% and 8.3% meaning 43 and 47 genes, respectively. Most importantly, for our purposes at least, *Cdh2* was identified as a strongly linked gene in one of the datasets. Interestingly, the *ApoE* gene, previously implicated in ASD and sporadic Alzheimer’s disease (AD; Won et al., 2013) was also identified in both datasets as a candidate gene. In fact, *ApoE* mutations could interfere with both developmental and synaptic CDH2 functions. First, APOE can significantly inhibit the binding of Reelin to its receptors, VLDLR and APOER2 (D’Arcangelo et al., 1999). This could impair Reelin-dependent, CDH2-mediated terminal translocation of migrating neuron precursors to the cortical plate. Synaptic functions of both Reelin and CDH2 are probably also very important since the appearance of ASD symptoms coincides with the synaptic development stage. ASD manifestation is primarily mediated by the synaptic roles of *ApoE* interfering with NMDA and AMPA receptor function. It is conceivable, however, that similarly to development, Reelin signaling disturbed by ApoE mutation contributes to the disease symptoms. This is supported by the importance of CDH2 in AMPAR trafficking (Nuriya and Huganir, 2006; Saglietti et al., 2007; Zhou et al., 2011) and the fact synaptic effects of ApoE mutations are transmitted *via* PI3K-Akt signaling pathways which is also essential in mediating the neural stem cell maintenance function of CDH2 during cortical development (Zhang Y. et al., 2010; Zhang et al., 2013).

In this regard, it is also interesting to note that *Cdh8, Cdh9, Cdh10* and *Cdh11*, four types of classic cadherins which are also expressed in the developing cortex, have also been linked to ASD recently indicating a potential, functional redundancy with *Cdh2* (Wang et al., 2009; Pagnamenta et al., 2011; Lefkovics et al., 2012). In addition, CDH13, a unique GPI-anchor-bound cell surface cadherin has been demonstrated to be a very important genetic factor in both ADHD and autism indicating that genetic redundancy and possibly molecular interactions between these proteins should be examined more closely in the future (Rivero et al., 2015).

### Peter’s anomaly (PA)

PA is a disease that is characterized by the failure of separation of the iris and the cornea (type 1) or the cornea and the lens (type 2) during eye development. Besides these, in about 70% of the patients who have bilateral manifestation additional symptoms including abnormalities in craniofacial and genito-urinary development, brachydactyly and short stature are also present (Bhandari et al., 2011). More importantly, a recent study identified a *de novo* heterozygous splicing variant of *Cdh2* in patients which would result in a truncated variant of the protein terminating translation at the first EC domain. Significantly, all 4 affected patients also displayed agenesis of the corpus callosum and cognitive delay which could be due to disturbance of axon guidance and synaptic maintenance, respectively, processes CDH2 is essential for (Reis et al., 2020).

Next, we discuss several diseases which are indirectly caused by CDH2 malfunction due to mutations in other proteins regulating its availability or function.

### Warburg Micro syndrome

Warburg Micro syndrome (WARBM) is a recessive autosomal developmental disorder, characterized by microcephaly, corpus callosum abnormalities, intellectual disability, optic atrophy and hypogenitalism (Morris-Rosendahl et al., 2010). Based on genetic screening, WARBM is classified into four different types: type 1 is caused by the inactivating mutation of *Rab3gap1* gene coding for a catalytic subunit of a GTPase-activating protein, (41% of the cases); type 2 is associated with the mutation in *Rab3gap2* gene (7% of the cases), type 3 mutation affects the *Rab18* gene (5%) and the loss-of-function mutation in the GTPase-activating protein TBC1D20 is responsible for the type 4 (Morris-Rosendahl et al., 2010; Handley et al., 2013; Liegel et al., 2013). All these mutations are affecting various members of the RAB-mediated intracellular transport pathways. Interestingly, however, these diseases share common symptoms with the *Cdh2* mutation linked ACOG syndrome described above which points to the direction that the inadequate vesicular turnover of CDH2 might also be responsible for WARBM. In fact, Wu and colleagues using a combination of *in vivo* and *in vitro* models showed direct evidence that the loss-of-function mutation in *Rab18* gene increases the lysosomal degradation of CDH2, which leads to its decreased membrane presence and caused migration defect and neurite growth (Wu et al., 2016). Furthermore, mouse *Cdh2* loss-of-function experiments resulted in premature differentiation of neuronal progenitors which led to decreased number of neurons in the cortex which might also contribute to the observed microcephaly in WARBM (Zhang J. et al., 2010; Wu et al., 2016; Khalesi et al., 2021).

### Miller-Dieker syndrome

Miller-Dieker lissencephaly syndrome (MDLS) is characterized by a combination of classic lissencephalic features (e.g., pachygyria) with microcephaly, intellectual disability, craniofacial malformation and seizures. It is caused by heterozygous deletion of chromosome 17p13.3 including the *Pafah1b1* (more commonly known as *Lis1*) gene which is responsible for the lissencephalic features and the *Ywhae8* gene (14.3.3e) gene the product of which interacts with proteins like CDC25A and CDC25B involved in the proper execution of mitosis. Mice with a heterozygous inactive allele of *Pafah1b1* have severe cortical and cerebellar lamination defects due to defective neuron migration (Hirotsune et al., 1998; Gambello et al., 2003). This is carried out through the regulation of dynein function *via* NUDEL and CDK5/p35 (Niethammer et al., 2000; Sasaki et al., 2000). Interestingly, in the same year, CDK5/p35 has also been established as the regulator of CDH2-mediated adhesion between cortical neurons (Kwon et al., 2000). Despite this, and the *in vivo* evidence pointing to CDH2 as the main regulator of cortical stemness *via* the β-catenin pathway, Cdh2 could not be linked genetically to MDLS before (Zhang J. et al., 2010; Zhang et al., 2013). Recently, however, Ladewig and colleagues (Iefremova et al., 2017) using a cortical organoid model grown from MDSL patients have demonstrated that alterations in the architecture of the cortical neurogenic niche due to disruption of the tubulin cytoskeleton also results in defective CDH2 - AKT - β-catenin signaling. This in turn, evokes an increased premature cell cycle exit and differentiation of cortical progenitors which is one of the main factors causing microcephaly.

Recently, an interesting paper examined the HIC1 (Hipermethylated in cancer 1) tumor suppressor protein which is also associated with MDS (Ray and Chang, 2020). Just like *Pafah1b1*, the *Hic1* gene is also included in the chromosomal deletion responsible for MDS (Carter et al., 2000). Ray and colleagues used the Xenopus as a neural crest model animal to study the effect of Hic knockdown. They found that Hic1-loss caused defective craniofacial development which was due to the abnormal migration of neural crest cells. Furthermore, they established that Hic1 regulates the expression of Cadherins 1, 2 and 11 *via* Wnt signaling. The role of CDH2 in the collective migration of the neural crest has been detailed above and these data when combined present a strong argument for the involvement of CDH2 both in the neural and craniofacial aspects of the Miller-Dieker syndrome.

### Periventricular nodular heterotopia (PVH)

We have to make one exception in this review despite the fact, that no direct genetic evidence implicates CDH2 involvement in this disease. Cortical malformations are a large group of neurodevelopmental diseases with a wide range of symptomatic appearances (Juric-sekhar and Hevner, 2019). Nevertheless, most of these are caused by the defective function of two extremely important processes during cortical development, namely progenitor proliferation and neuron precursor migration. Here, we focus on periventricular heterotopia which occurs when a large number of neurons and proliferative progenitors fail to migrate normally and accumulate close to the ventricle. Furthermore, disruption of the apical adherens junction barrier sometimes allows this cell mass to intrude into the ventricle. Notably, a very similar process takes place in *Cdh2-/-* mutants (Gil-Sanz et al., 2014). Initial genetic studies identified that mutations in *FilaminA* (*Flna*) and *Arfgef2* genes are associated with the disease. FLNA is an actin regulatory protein promoting branching at the leading edge of migrating cells (Gorlin et al., 1990; Flanagan et al., 2001). It has been also shown to be required for cell-to-cell contact formation during heart and vessel development (Feng et al., 2006). It is an X-chromosome-linked gene and its mutation causes PVH in heterozygous females but lethality in hemizygous males (Hart et al., 2006). Interestingly, postmortem examination of PVH patients led to the conclusion that the cells forming the nodules are composed of late-born neurons (Ferland et al., 2009). In addition, they also described strong denudation of the ventricular wall a feature phenocopied in mice in which CDH2 function was disrupted perinatally (Oliver et al., 2013). These similarities make it very tempting to speculate that loss-of *Flna* which disrupts the actin-based cytoskeleton eventually also leads to CDH2-based adherens junction disruption producing intraventricular heterotopia.

## CDH2 and its connection to neurodegenerative diseases

### Dementia and neurodegenerative diseases

With increasing life expectancy all around the world but particularly in developed countries, dementia presents an extremely large social and economic burden on society. In 2018, approximately 9 million people, representing about 7% of the population aged over 60 are living with dementia in EU member states, up from 5.9 million in 2000, and this number is projected to increase to around 14 million in 2040 (OECD/EU (2018). according to the recent report, Health at a Glance: Europe 2018: State of Health in the EU Cycle, OECD Publishing, Paris. https://doi.org/10.1787/health_glance_eur-2018-en).

### Alzheimer’s disease

Alzheimer’s disease is the most common form of frontotemporal dementia characterized by severe neurodegeneration and cortical atrophy. In 2019, Alzheimer’s Disease International estimated that there were over 50 million people living with dementia globally, a figure set to increase to 152 million by 2050, and the current annual cost of dementia is estimated at US $1trillion, a figure set to double by 2030. Alzheimer’s Disease International (2019). Synaptic loss is one of the earliest features of AD which correlates well with its symptomatic progression. Generally, it is caused by the extracellular deposition of the toxic amyloid-β and the intracellular accumulation of phosphorylated tau protein tangles resulting in the disruption of neuronal circuits in the prefrontal cortex, hippocampus and at later stages in other brain areas as well (Deture and Dickson, 2019). Synaptic loss, however, can occur independently of amyloidosis *via* enhanced microglial phagocytosis of synaptic structures (Rajendran and Paolicelli, 2018; Subramanian et al., 2020). Below we comprise some of the data which indicates potential involvement of CDH2 in the etiology of AD.

### CDH2, presenilin and the early-onset familial Alzheimer’s disease

Familial Alzheimer’s disease (FAD) consists only 5% of the total AD cases worldwide. Mutations responsible for FAD occur in 3 genes: amyloid precursor protein (APP), presenilin 1 and 2 (PSEN1 and 2, respectively). All three proteins have their specific synaptic function, including but not limited to neuronal outgrowth, vesicular cycle and release, lysosomal homeostasis and autophagy (Elder et al., 1996; Hung and Livesey, 2018; Padmanabhan et al., 2021).

PSEN1 is the proteolytic subunit of the γ-secretase complex which cleaves transmembrane proteins by initiating a wide range of indispensable biochemical processes and pathways. However, this complex also cleaves the transmembrane domain of the APP protein which produces the toxic and accumulative form of the amyloid-β-peptide (Aβ1-42) forming the characteristic plaque depositions in Alzheimer’s disease (Takasugi et al., 2003; Watanabe et al., 2021). Besides its plasma membrane localization, PSEN1 is also present in the endoplasmic reticulum, where it is responsible for post-translational endoproteolytic processing by cleaving the N- or C-terminal fragments (NTF or CTF, respectively) of various proteins (Wolfe, 2019). Not surprisingly, the loss of PSEN1 function leads to irreversible malfunctions. PSEN1 knock-out mice die perinatally (Shen et al., 1997) while conditional ablation of the gene in proliferating neuronal progenitors disrupts both neurogenesis and cell proliferation (Kim and Shen, 2008). Human mutations in PSEN1 gene, however, do not replicate the phenotype of the knockout models, moreover, they can rescue the full knock-out lethality (Qian et al., 1998). Currently, more than 350 PSEN1 human mutations are known which affect the enzymatic function of the protein^1^, and most of them lead to neuronal cell death and cytotoxicity (Edwards-Lee et al., 2006; Lazarov et al., 2006).

In 1999, Georgakopoulos and colleagues showed for the first time, that PSEN1 can form an intracellular macromolecular complex with cadherins and catenins which maintains cell-to-cell connections and neurite outgrowth. This was also confirmed by electron microscope using rat cornea samples where PSEN1 and CDH2 co-localized in synaptic junctions (Georgakopoulos et al., 1999). Furthermore, utilizing recent developments in super-resolution microscopy, namely Stimulated Emission Depletion (STED) and Single-Molecule Localization Microscopy (SMLM), it has been demonstrated at an even greater resolution that the whole γ-secretase complex is in close proximity to the CDH2-based macromolecular hub (Escamilla-Ayala et al., 2020). It has also been described that exclusively PSEN1, and not PSEN2 regulates CDH2 levels by cleaving the cytoplasmic part of the protein (Jang et al., 2011) and thereby also altering the vesicular trafficking of CDH2 from the ER to the plasma membrane (Uemura et al., 2003). This precise regulation might work as an ultimate pro-survival signal, as loss of homophilic CDH2-binding due to *Psen1* mutation and/or malfunction leads to synapse loss and cell death. Further substantiates this notion that C-terminal cleavage of CDH2 by PSEN1/γ-secretase complex produces a truncated CTF which in turn alters AMPA-mediated synaptic transmission and amplifies the effect of toxic Aβ-induced synapse damage (Priller et al., 2007; Jang et al., 2011; Andreyeva et al., 2012). Beyond the toxic Aβ accumulation in the AD brain, truncated CDH2 C-terminal fragments also form aggregates in human AD patients (Andreyeva et al., 2012). Interestingly, performing a high-throughput genome-wide analysis revealed a rare autosomal copy number (488kb) polymorphism in the *Cdh2* gene in FAD patients reinforcing the idea that PSEN1/CDH2 signaling is an important part of AD synaptic pathogenesis (Hooli et al., 2014).

### Sporadic Alzheimer’s disease, ApoE, Reelin and CDH2

Sporadic or late-onset AD (LOAD) cases are the most common forms of the disease which usually appear after the age of 60. There are several risk genes which increase the likelihood of developing LOAD, the most important of them is the lipid carrier protein *Apoliporotein E* (*ApoE*). The *ApoE* gene has 3 slightly different alleles in humans *ApoE*_2_, *ApoE*_3_ and *ApoE*_4_. The last one has a strong correlation with developing LOAD (Husain et al., 2021) while *ApoE3* is neutral and *ApoE2* has a positive anti-LOAD effect. All APOE protein variants are ligands for both the low- (LDLR) and the very low-density lipoprotein receptors (VLDLR) as well as ApoE receptors (reviewed in Bock and May, 2016). These receptors are mostly localized in the postsynaptic density and interact with glutamatergic receptors, as well as adhesion and scaffold proteins. This means that Reelin and APOE proteins share a signaling pathway (or at least the receptors) in the synapse. In addition, APOE4 also influences vesicular turnover and receptor recycling leading to altered Reelin and glutamate signaling (Lane-Donovan and Herz, 2017). Interestingly, Reelin also interacts with APP by regulating its localization at the plasma membrane and promotes dendritic arbor development (Hoe et al., 2009). This and other results highlight that Reelin signaling can be neuroprotective in AD at the early stages. Increased levels of Reelin in human AD CSF appear at the early stages of the disease which might be the result of disrupted ApoER signaling or a compensatory mechanism to protect synapses (Sáez-Valero et al., 2003; Botella-López et al., 2006; Lopez-Font et al., 2019). In contrast, at later stages increasing the amount of Reelin depositions also correlates well with reduced memory formation in aged wild-type rodents indicating a potential dual function for the protein in AD. This phenomenon is greatly enhanced both in AD mouse model and human LOAD cases, where Reelin deposits are co-localized with Aβ plaques, fibrillary tangles and correlate with cognitive deficits showing a distinct spatial and temporal function of Reelin in AD pathogenesis (Knuesel et al., 2009; Ramsden et al., 2022).

Considering the cooperation between Reelin signaling and CDH2 through the small GTPase RAP1, it is quite tempting to suggest that CDH2-based synaptic junctions are affected in LOAD. Accordingly, the deposition of Aβ plaques decreases the surface level of CDH2 *via* phosphorylation of Tau and p38 MAPK signaling (Ando et al., 2011). Furthermore, by measuring CDH2 degradation product levels in brain homogenates and CSF from human AD patients Choi et al. (2020) showed that its C-terminal fragment is accumulated in the brain parenchyma. In parallel, NTF levels are also elevated in both human and rodent CSF. Based on this, we dare to hypothesize that CDH2 is a significant downstream effector of the Reelin-ApoER2/VLDLR-DAB pathway not only during development but also in the adult brain. Moreover, the abovementioned evidence suggests that Alzheimer’s disease-related ApoE-mutations can disrupt neural function at least partly by interfering with CDH2-dependent synaptic plasticity.

### Huntington disease

Huntington disease (HD) is an autosomal fatal neurodegenerative disorder which manifests in progressive chorea, motor disfunction and dementia caused by the CAG expansion repeat of the *huntingtin* gene (HTT). 36 or more CAG repeats change the structure of the mutated HTT (mHTT) protein which will develop soluble monomers and oligomers, then gather as mHTT fibrils eventually causing large inclusions along the cells. This leads to cellular toxicity and cell death of the striatal neurons which also spreads to other brain areas (Gallardo-Orihuela et al., 2019; Tabrizi et al., 2020). Although the disease is usually diagnosed in mid-age, more and more evidence support the notion that HD is a developmental disease and mHTT presence already affects perinatal brain development and wiring. The first indication of its developmental function was when Zeitlin and colleagues demonstrated that full elimination of the *Htt* gene causes early embryonic lethality and elevated levels of cell death (Zeitlin et al., 1995). Loss of *Htt* during cortical development resulted in a decrease of progenitor and an increase in postmitotic cell numbers indicating a premature cell cycle exit (Molina-Calavita et al., 2014). Later, a knock-in model expressing an HTT protein with an increased poly Q region (Q111) featured delayed cell cycle exit supporting this notion (Molero et al., 2016). Moreover, researchers also found subpallial periventricular heterotopias and misplaced cells in this model (Arteaga-bracho et al., 2016). These results highlight the fact that HTT protein is an important molecular player during brain development, which CDH2 is also involved in. Recently, the first direct functional connection between HTT and CDH2 during cortical development was provided by Barnat et al. (2017). Generating dorsal telencephalon-specific loss-of *Htt* restricted to postmitotic cells, they showed that HTT is indispensable for normal multipolar/bipolar transition during radial migration in the embryonic cortex. Moreover, they also found that HTT affects CDH2 localization through regulation of its Rab11-dependent endosomal trafficking. The reintroduction of Rab11 in animals expressing mHTT proteins could prevent the migration deficit and the mislocalization of CDH2. This interesting connection between HTT and CDH2 was also supported by another study examining the cortical development of 13 weeks old human fetuses carrying polyQ HTT mutation. CDH2-based adherens junction complexes were disrupted at the bottom of the ventricular zone, which leads to abnormal polarization, cell production and fate commitment (Barnat et al., 2020). Very recently, a study analyzing postmortem human tissue from patients with Huntington disease revealed that the abovementioned developmental malformations affected the adult brain, in fact, as changes were still recognizable in adulthood. Examining 8 individuals, they found periventricular heterotopias all along the ventricles, which might be a clear representation of CDH2 function loss *via* mHTT in HD patients (Hickman et al., 2021). Since CDH2 has multiple functions during brain development, it is not surprising that mHTT influences synaptic physiology by altering CDH2-based synaptic junctions. Previously, we highlighted the functional importance of the fine balance between ADAM10 metalloprotease and CDH2 expression levels, which is also completely altered in HD. It has been shown that mHTT triggers the postsynaptic accumulation of ADAM10 which in turn leads to the sequestration and cleavage of CDH2. Furthermore, pharmacological inhibition of ADAM10 could prevent the proteolysis of CDH2 and improve the electrophysiological properties of striatal neurons (Vezzoli et al., 2019). Therefore, just like ADAM10, CDH2 could also be an interesting therapeutics target for slowing or halting the disease in the early phase.

### Huntington disease, CDH2, and the hydrocephaly connection

In hydrocephaly (HC) the ventricles of the brain are abnormally enlarged (megaloventriculi) and filled with cerebrospinal fluid. HC can occur due to genetic causes (congenital HC; (Abdelhamed et al., 2018; Furey et al., 2018; Roy et al., 2019) or as a secondary symptom due to external factors like intraventricular hemorrhages or various tumors (acquired HC) (Kahle et al., 2016; Castaneyra-Ruiz et al., 2018). The elevated pressure caused by the fluid build-up in the brain can cause serious damage to the surrounding brain tissue, particularly in the ependymal cell lining of the ventricles which are important in promoting CSF circulation. Blockage within the CSF draining system or conditions disrupting cilial structure or coordinated cilial movements also results in hydrocephaly which in turn, leads to the loss of the ependymal lining (denudation) of the ventricle walls (Tissir et al., 2010; Abdelhamed et al., 2018). Ependymal cells are derived from radial glia progenitor cells (Spassky, 2005), and consequently, they also do not have tight junction connections. Instead, they are held together exclusively by AJs and gap junctions (Del Bigio, 1995). As described beforehand, CDH2 is an integral part of AJs in the developing CNS so it was not surprising, when it was found that the neurogenic SVZ niche was disrupted in the hydrocephaly model *Hyh* mutant. Causality between the two phenomena was established when later experiments demonstrated that loss-of CDH2-based adherens junctions dispersed ependymal cells of the mouse brain resulting in ventricular wall denudation and hydrocephaly (Gate et al., 2012; Oliver et al., 2013; Guerra et al., 2015). Coincidentally, loss of AJs also precedes ependymal denudation in human fetuses with spina bifida aperta (Sival et al., 2011) which correlates well with the fact that mouse loss-of-function model of CDH2 also has partial neurulation defects (Luo et al., 2001). Moreover, congenital hydrocephalus caused by loss of the *Htt* gene in a mouse model was also associated with corpus callosum defects (Dietrich et al., 2009) in which CDH2 is also heavily involved (see above). The explanation for these events can be found in the normal function of the HTT protein which is to regulate the ciliogenesis of ependymal cells (Keryer et al., 2011). As a consequence, late-onset Huntington disease patients have been misdiagnosed before as normal pressure hydrocephalus (Caserta and Sullivan, 2009; Dennhardt and Ledoux, 2010). Interestingly, there is a form of congenital HC in which intraventricular pressure remains normal called idiopathic normal pressure hydrocephalus (iNPH) also characterized by gait disturbance and dementia. Due to similar symptoms and decreased CSF Aβ1 – 42 levels, iNPH can be mistakenly diagnosed as Alzheimer’s disease (Picascia et al., 2015; Schirinzi et al., 2015).

From the evidence discussed above and in previous parts of this review indicating the involvement of CDH2 in both Alzheimer’s disease and Huntington disease, we dare to hypothesize that CDH2 might be a common pathological factor during the development of these diseases.

In summary, CDH2-loss-dependent ventricular wall denudation leads to CSF circulation defects, and vice versa, CSF circulation defects can result in ventricular wall denudation creating a positive feedback phenomenon that leads to HC. Either way, CDH2 is right in the middle of this process.

## Future considerations

In this review, we summarized recent developments which furthered our understanding of the function of CDH2 in neural development and disease. For years, people working on this protein have wondered how it was possible that CDH2 did not have any direct evidence linking it to any neural diseases. Well, accumulating results of the last few years have put this protein into an entirely new perspective and undoubtedly, the coming years will provide even more novel aspects regarding CDH2 function. Certainly, one of the most interesting question is how one gene could be involved in so many diseases. Well, the most likely answer lies in the occurrence of somatic mutations affecting different areas of the brain at various developmental stages. Therefore, the clarification of whether inherited or somatic mutations are behind a given disease, is essential. And if somatic mutations are responsible then establishing at what stages and which areas are affected will be of utmost importance. In some NDs the overwhelming percentage of the cases are caused by sporadic somatic mutations (e.g., Alzheimer’s disease) therefore further large-scale sequencing efforts will have to be carried out in the future which will help to reveal the genetic and molecular interactions behind these diseases. The next important task concerns the regulation of CDH2 levels. Is there a functional reason behind the choice of endocytic vs. shedding method of regulation, or are these complementary or alternative to each other in different cell types or biological processes? Finally, a very interesting and potentially clinically relevant issue is whether shed extracellular CDH2 fragments in the CSF could serve as detectable disease markers in various implicated diseases affecting the CNS.

Although this review focuses on CDH2, the research perspectives section should not ignore the question of potential functional redundancy and possible heterodimerization between CDH2 and other classic cadherins. There are a lot of other classic cadherins which are also expressed strongly in the developing mammalian CNS (Ranscht and Dours-Zimmermann, 1991; Bekirov et al., 2002; Takahashi and Osumi, 2008; Mayer et al., 2010; Lefkovics et al., 2012) some of which have been linked previously to diseases causing various forms of intellectual disability (for review see Redies et al., 2012; Hawi et al., 2018). With the continuous advancement of the CRISPR/Cas9 technique, researchers finally have a straightforward and relatively cheap tool to investigate the question of redundancy among various classic cadherins by creating double or when it is possible, various combinations of multiple mutations. Undoubtedly this will help to further the efforts of our common goal in developmental neuroscience: understanding the formation of the human brain.

## Author contributions

ZIL and ZL planned and equally provided an intellectual contribution during manuscript preparation including conceiving the idea, collecting information, and writing the manuscript. Both authors approved it for publication.

## Footnotes

1
https://www.alzforum.org/mutations/
